# A novel DNA methylation‐driver gene signature for long‐term survival prediction of hepatitis‐positive hepatocellular carcinoma patients

**DOI:** 10.1002/cam4.4838

**Published:** 2022-05-30

**Authors:** Jie Fu, Wei Qin, Qing Tong, Zhenghao Li, Yaoli Shao, Zhiqiang Liu, Chun Liu, Zicheng Wang, Xundi Xu

**Affiliations:** ^1^ Department of General Surgery The Second Xiangya Hospital of Central South University Changsha China; ^2^ Department of General Surgery South China Hospital of Shenzhen University Shenzhen China

**Keywords:** DNA methylation‐driver gene, hepatitis‐positive HCC, nomogram, prognosis, risk score

## Abstract

**Background:**

Abnormal DNA methylation is one of the most general epigenetic modifications in hepatocellular carcinoma (HCC). Recent research showed that DNA methylation was a prognostic indicator of all‐cause HCC and nonviral HCC. However, whether DNA methylation‐driver genes could be used for predicting survival, the probability of hepatitis‐positive HCC remains unclear.

**Methods:**

In this study, DNA methylation‐driver genes (MDGs) were screened by a joint analysis of methylome and transcriptome data of 142 hepatitis‐positive HCC patients. Subsequently, a prognostic risk score and nomogram were constructed. Finally, correlation analyses between the risk score and signaling pathways and immunity were conducted by GSVA and CIBERSORT.

**Results:**

Through random forest screening and Cox progression analysis, 10 prognostic methylation‐driver genes (AC008271.1, C11orf53, CASP8, F2RL2, GBP5, LUCAT1, RP11‐114B7.6, RP11‐149I23.3, RP11‐383 J24.1, and SLC35G2) were screened out. As a result, a prognostic risk score signature was constructed. The independent value of the risk score for prognosis prediction were addressed in the TCGA‐HCC and the China‐HCC cohorts. Next, clinicopathological features were analyzed and HBV status and histological grade were screened to construct a nomogram together with the risk score. The prognostic efficiency of the nomogram was validated by the calibration curves and the concordance index (C index: 0.829, 95% confidence interval: 0.794–0.864), while its clinical application ability was confirmed by decision curve analysis (DCA). At last, the relationship between the risk score and signaling pathways, as well as the correlations between immune cells were elucidated preliminary.

**Conclusions:**

Taken together, our study explored a novel DNA methylation‐driver gene risk score signature and an efficient nomogram for long‐term survival prediction of hepatitis‐positive HCC patients.

## Graphical abstract

DNA methylation‐driver genes (MDGs) were screened by a joint analysis of methylome and transcriptome data of 142 hepatitis‐positive HCC patients. A prognostic risk score and nomogram were constructed based on these MDGs and clinicopathological features.

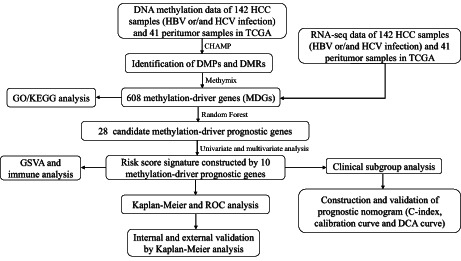

## 1 INTRODUCTION

Liver cancer is an important fatal cancer type worldwide, and the majority of liver cancer is hepatocellular carcinoma (HCC).^1^ The most common causes of HCC include hepatitis infection, excessive drinking, aflatoxin, nonalcohol fatty liver disease (NAFLD), excess body weight, and type 2 diabetes.^1^, ^2^, ^3^ Although the treatment of HCC is gradually enriched, the prognosis is still unsatisfactory.^4^, ^5^, ^6^ In this condition, it is urgent to seek effective targets for the treatment and prognosis prediction of HCC.

DNA methylation is a crucial epigenetic modification type participating a series of biological and pathological processes.^7^, ^8^, ^9^, ^10^, ^11^ Aberrant DNA methylation states have been identified in numerous malignant diseases, such as breast cancer, melanoma, colorectal cancer, prostate cancer, and gastric cancer.^7^, ^12^, ^13^, ^14^, ^15^, ^16^, ^17^ Meanwhile, recent studies showed that DNA methylation probes, as well as DNA methylation‐driven genes (MDGs), can be used in the prediction of prognosis, diagnosis and treatment of diseases.^12^, ^18^, ^19^, ^20^, ^21^ Therefore, systematically clarifying the DNA methylation status of cancer may provide a new idea for improving the prognosis of patients.

Alterations of epigenetic status have been widely identified in HCC, such as histone modification, DNA methylation and m6A.^22^ In recent years, DNA methylation status has been reported participated in the process of hepatocarcinogenesis in many ways, such as transcriptional regulation and oncogenic dedifferentiation.^23^, ^24^ Furthermore, recent researches have showed that DNA methylation can used for prognosis prediction and treatment of HCC.^25^, ^26^ In addition, the prediction models constructed by DNA methylation probes or MDGs also showed good prediction efficiency in all‐cause induced HCC and nonviral HCC.^27^, ^28^, ^29^, ^30^ Considering that HCC developed from hepatitis has different pathogenesis and pathological characteristics from HCC caused by other causes, we studied whether MDGs can be used for prognosis prediction of hepatitis‐positive HCC.

In this study, methylome and transcriptome data were comprehensively analyzed and 10 prognostic MDGs (AC008271.1, C11orf53, CASP8, F2RL2, GBP5, LUCAT1, RP11‐114B7.6, RP11‐149I23.3, RP11‐383 J24.1, and SLC35G2) were screened out. As a result, a novel risk score signature and an efficient nomogram for survival prediction of hepatitis‐positive HCC were constructed. Moreover, the functional and immune characteristics of different risk groups were preliminarily clarified. These results revealed potential intervention targets to further improve the prognosis of hepatitis‐positive HCC patients.

## 2 MATERIALS AND METHODS

### 2.1 Download data from public databases

DNA methylation data (Illumina Infinium Human Methylation450 platform) and expression data in fragments per kilobase million (FPKM) form of HCC patients were retrieved from The Cancer Genome Atlas (TCGA) database. The corresponding detailed clinical data were acquired from cBioPortal (TCGA‐HCC cohort). Next, 159 Chinese HCC patients with hepatitis infection from the Zhongshan Hospital of Fudan University were used as an external validation cohort (China‐HCC cohort),^31^ and the corresponding FPKM data were retrieved from NODE database.

### 2.2 Preprocessing of RNA‐sequencing and DNA methylation data

All of the FPKM data were converted to transcripts per kilobase per million (TPM) data before subsequent processing. Inclusion criteria in this study: 1. Overall survival (OS) time >1 month; 2. Patients were infected with HBV) and/or HCV. Ultimately, 142 HCC samples and 41 peritumor samples (TCGA‐HCC cohort) containing methylome and transcriptome data were included in this study. For DNA methylation data, methylation probes in the Illumina HumanMethylation450 platform with “NA” results were deleted, and 380,828 methylation probes remained for subsequent analyses.

### 2.3 Identification of differentially methylated probes (DMPs), differentially methylated regions (DMRs) and MDGs

Differential methylation analysis was performed between 142 HCC samples and 41 peritumor samples from TCGA by the “ChAMP” package in R.^32^ After the quality control and normalization processes, DMPs and DMRs were identified. Subsequently, methylome and transcriptome data were analyzed synthetically by the “MethylMix” package for the identification of MDGs.^33^

### 2.4 Functional enrichment analyses

Gene Ontology (GO) and Kyoto Encyclopedia of Genes and Genomes (KEGG) pathway analyses were performed with the MDGs using the “clusterProfiler” package.^34^

### 2.5 Risk score construction and survival analyses

Survival‐related MDGs were screened by the random forest method (ntree = 100) using the “survivalsvm” and “randomForestSRC” packages.^35^ Next, these survival‐related MDGs were analyzed by the univariate Cox method using the “survival” package, and the prognostic genes (*p* values <0.05) were further analyzed by the multivariate Cox regression method. After that, a prognostic risk score was constructed by the 10 most significant prognostic MDGs (*p* value <0.05) and their efficiencies. Patients were divided into two risk groups according to the median value of the risk score, and prognostic values were identified by survival analyses using the “ggrisk”, “survival” and “timeROC” packages.

### 2.6 Validation of the MDGs‐based risk score signature

TCGA‐HCC patients were separated into two internal validation subgroups according to the presence or absence of HBV infection: the HBV group (91 patients only infected with HBV) and the non‐HBV group (44 patients with HCV infection only and 7 patients with HBV and HCV infection). The subgroups of the TCGA‐HCC patients, as well as China‐HCC patients, were used for the evaluation of prognostic value by Kaplan–Meier plotter analysis.

### 2.7 Preprocessing and analysis of clinicopathological features

Clinicopathological features with a missing rate of less than 10% from the TCGA‐HCC cohort were analyzed in our study, including AFP level (“< 400”, “≥ 400”), age (“< 55”, “≥ 55”), gender (“female”, “male”), HBV status (“HBV”, “non‐HBV”), histological grade (“G1‐G2”, “G3‐G4”), race (“Asian”, “non‐Asian”), surgical margin (“R0”, “R1‐R2”), T stage (“T1‐T2”, “T3‐T4”) and vascular invasion status (“None”, “Macro–Micro”). Missing values were automatically interpolated by the “mice” package. Next, prognostic values of these enrolled clinical features and risk score were evaluated by univariate Cox regression analysis in TCGA‐HCC cohort. Risk factors with *p* values <0.05 (HBV status, histological grade, race, T stage, vascular invasion status and risk score) were then included in the multivariate regression analysis. After that, risk factors with *p* values <0.1 (HBV status, histological grade and risk score) were screened out. Finally, subgroup analysis was performed using the “forestplot” package.

### 2.8 Prognostic nomogram construction and evaluation

Next, risk score signature and clinicopathological features (HBV status and histological grade) were used for the construction of a prognostic nomogram by the “rms” package in R. Prognostic efficiency of the nomogram was evaluated by concordance index (C index) and calibration curves, and the clinical application value was validated by decision curve analysis (DCA).

### 2.9 Gene set variation analysis (GSVA) and immune analysis

GSVA was conducted between the high‐ and the low‐risk group of TCGA‐HCC patients using the “GSVA” package.^36^ For immune analysis, immune cell components were analyzed by CIBERSORT, while correlation analysis of immune cells was analyzed using the “ggcorrplot” package.

### 2.10 Statistical analysis

All of the data were analyzed and visualized by R 4.1.0. The survival data were statistically analyzed by log‐rank test. The continuous variables between the two groups were compared by Student *t*‐test. *p* < 0.05 was considered statistically significant.

## 3 RESULTS

### 3.1 Identification of DMPs, DMRs and MDGs

**FIGURE 1:**
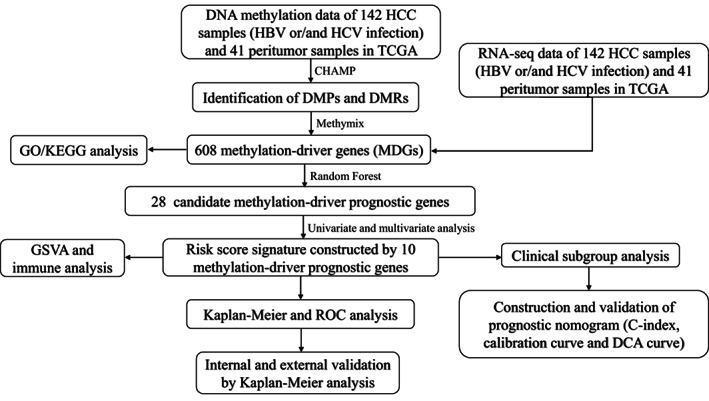
Analysis flowchart

**FIGURE 2:**
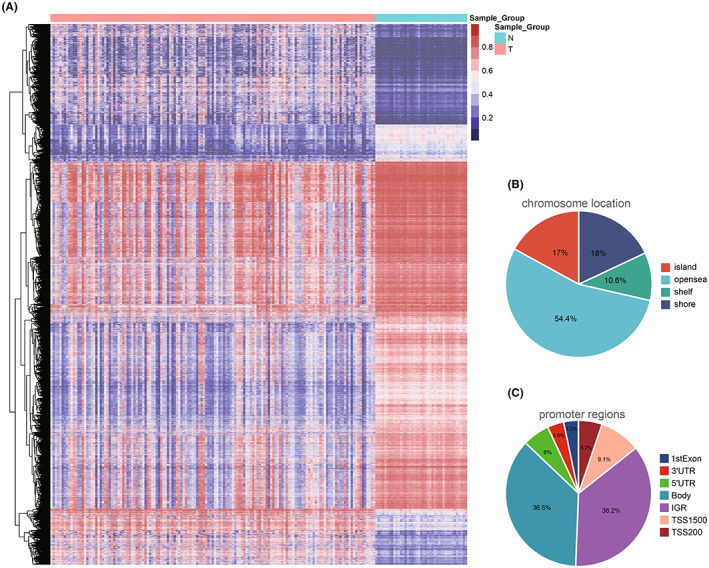
Identification of the DMPs between 142 HCC samples and 41 peritumor samples. (A) Heatmap of DMPs. (B) Pie chart of the DMPs based on their chromosome location. (C) Pie chart of the DMPs based on their promoter regions

The study flowchart is shown in Figure 1. First, the results of cluster analysis combined with the results of principal component analysis (PCA) indicated that tumor tissues and peritumor tissues can be distinguished well at the DNA methylation level (Figure S1 and Table S1). After that, DMPs and DMRs between 142 HCC samples and 41 peritumor samples from TCGA database were identified using the “ChAMP” package and visualized by heatmap (Figure 2A). In general, tumor tissues showed a more hypomethylated state than peritumor tissues. Next, 56,714 DMPs with adjust *p* < 0.05 and |Δβ| > 0.2 were picked out and visualized by pie chart based on their chromosome location (shelf, shore, island and open sea) (Figure 2B) or promoter regions (TSS1500, body, 1st exon, TSS200, 3′ UTR, IGR and 5′ UTR) (Figure 2C). To deeply clarify the changes of gene expression influenced by DNA methylation status, methylome and transcriptome data were integrated and analyzed, and 608 MDGs were identified.

### 3.2 Enrichment analysis of the 608 MDGs

**FIGURE 3:**
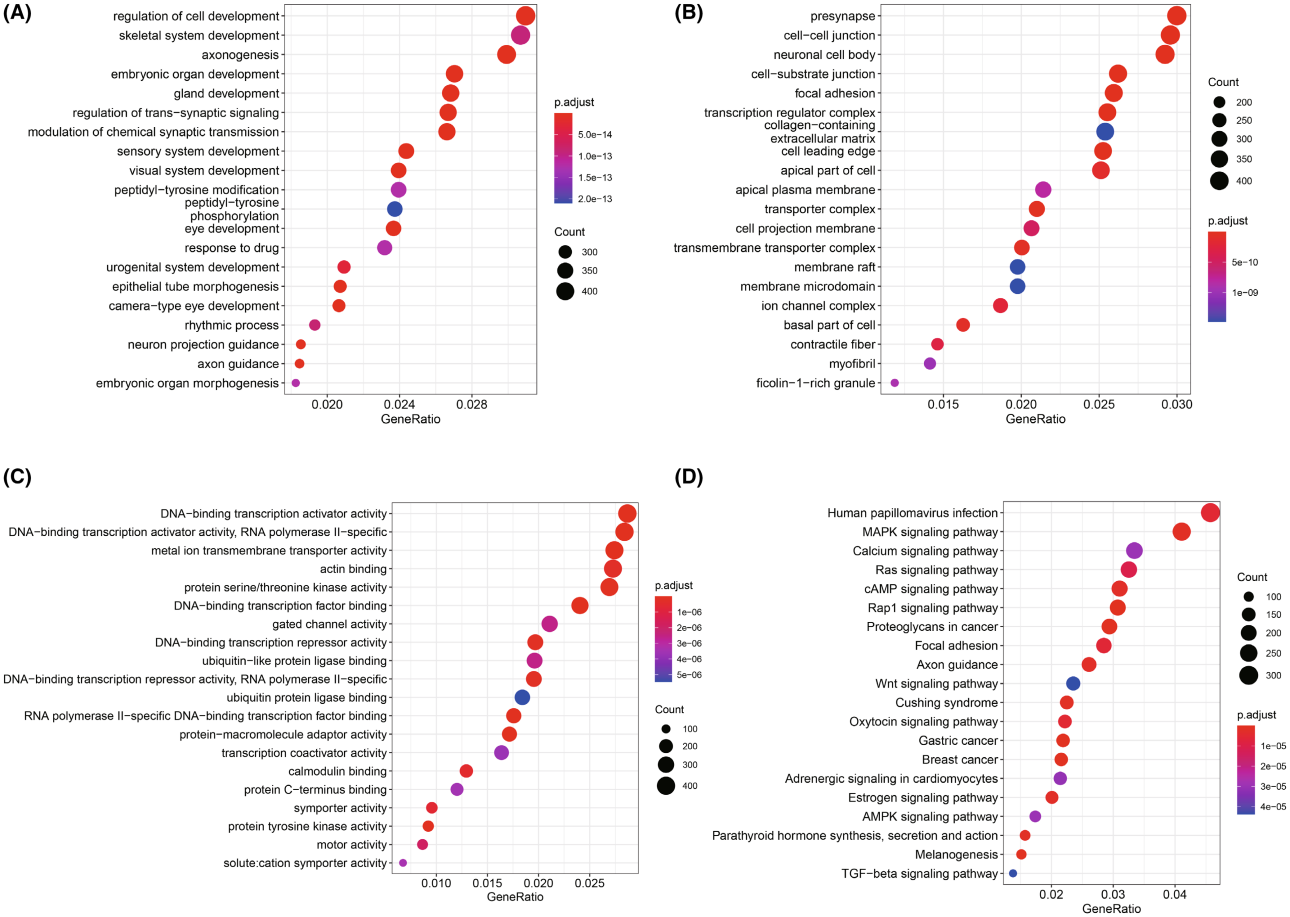
Function enrichment analysis of the 608 MDGs. (A) The results of BP analysis. (B) The results of CC analysis. (C) The results of MF analysis. (D) The results of KEGG analysis

To elucidate the potential functional roles of the 608 MDGs, GO and KEGG analyses were conducted. The results of biological process (BP) analysis suggested that MDGs were associated with the regulation of cell development, gland development, response to drug, gland development, etc (Figure 3A). MDGs were enriched in cellular components (CC), including cell–cell junctions, transcription regulator complexes, transporter complexes, cell projection membrane, etc (Figure 3B). MDGs were enriched in molecular function (MF), including activity of DNA‐binding transcription activator, activity of metal ion transmembrane transporter, activity of protein serine/threonine kinase, etc (Figure 3C). KEGG analysis results suggested that MDGs were associated with MAPK, calcium, Ras, cAMP, Wnt signaling pathways, etc (Figure 3D).

### 3.3 Risk score construction and evaluation

**FIGURE 4:**
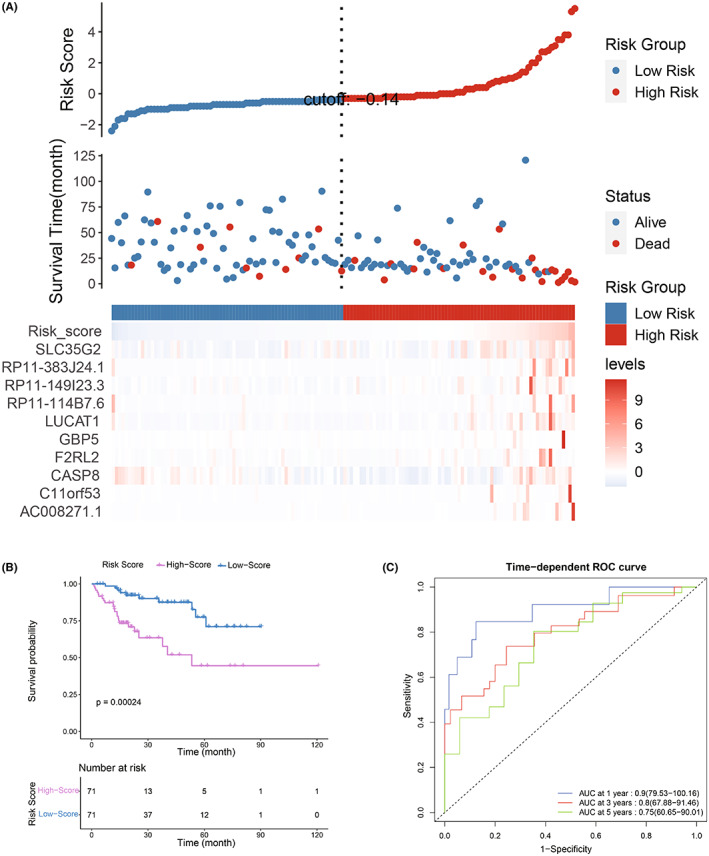
Construction and validation of the prognostic value of the risk score signature. (A) Cutoff value (upper panel), survival status (middle panel) and expression levels of risk score and 10 prognostic MDGs (lower panel). (B) Kaplan–Meier analysis result of the two groups divided by the cutoff value. (C) ROC curves of the risk signature

First, 28 survival‐related MDGs were screened by a random forest model. Next, these 28 MDGs were analyzed by a univariate Cox model and multivariate Cox regression model (Table 1). Next, a risk score was constructed by the remaining 10 prognostic MDGs (*p* value <0.05). The mixture models of these 10 MDGs were shown in Figure S2A. Notably, expression and methylation levels of all these 10 MDGs were significantly negatively correlated (Figure S2B). Risk score was calculated by the sum of the expression values of the 10 prognostic MDGs multiplied by their efficiencies: TPM value of AC008271.1 × 2.906368305 + TPM value of C11orf53 × 0.068665668 + TPM value of CASP8 × (−0.1420 29436) + TPM value of F2RL2 × 0.044918884 + TPM value of GBP5 × 0.008064013 + TPM value of LUCAT1 × 0.2312 47313 + TPM value of RP11‐114B7.6 × (−0.720255272) +  TPM value of RP11‐149I23.3 × 1.431387741 + TPM value of RP11‐383 J24.1 × 0.169909140 + TPM value of SLC35G2 × 0.137406119. HCC patients were separated into two groups (high‐ and low‐risk groups) according to the median risk score. Survival status, as well as the expression levels of the risk score and 10 prognostic MDGs, are presented in Figure 4A. K‐M plotter result showed that patients in the low‐score group had a significantly better prognosis than those in the high‐score group (Figure 4B). Subsequently, ROC curves of the risk score signature were visualized, with corresponding areas under the curve (AUCs) of 0.9, 0.8 and 0.75, respectively (Figure 4C). This result revealed the excellent prognostic efficacy of this model.

**TABLE 1:** Univariate and multivariate Cox regression analysis of the 28 MDGs

	Univariate analysis		Multivariate analysis	
Genes	HR (95% CI)	*p*	HR (95% CI)	*p*
ABCG5	0.99 (0.97–1.01)	0.192	NA	NA
AC008271.1	5.66 (2.2–14.55)	0***	40.04 (4.99–321.19)	0.001**
APCS	1 (1–1)	0.67	NA	NA
C11orf53	1.05 (1.02–1.07)	0***	1.09 (1.04–1.14)	0***
C5orf58	1.16 (1.05–1.28)	0.003**	1.31 (0.92–1.88)	0.137
CASP8	1.1 (1.03–1.17)	0.007**	0.79 (0.65–0.96)	0.018*
DCAF4L1	1.53 (1.17–2.01)	0.002**	2.26 (0.99–5.16)	0.053
F2RL2	1.07 (1.02–1.11)	0.002**	1.17 (1.04–1.31)	0.007**
FBXL22	0.83 (0.22–3.06)	0.777	NA	NA
FCER1G	1 (1–1.01)	0***	1 (1–1.01)	0.07
GBP5	1.01 (1–1.01)	0.007**	1.01 (1–1.02)	0.009**
KB.1991G8.1	1.84 (1.13–3)	0.014*	0.8 (0.21–3.03)	0.745
LUCAT1	1.07 (1.04–1.11)	0***	1.19 (1.01–1.41)	0.035*
MYBPHL	1.35 (1.09–1.67)	0.006**	1.25 (0.79–1.98)	0.345
RP11‐114B7.6	1.19 (1.08–1.3)	0***	0.38 (0.25–0.58)	0***
RP11‐1336O20.2	11.98 (0.31–467.63)	0.184	NA	NA
RP11‐149I23.3	2.07 (1.27–3.36)	0.003**	5.33 (1.7–16.71)	0.004**
RP11‐383 J24.1	1.1 (1.06–1.14)	0***	1.23 (1.08–1.41)	0.002**
RP11‐443P15.2	1.04 (1.02–1.06)	0***	1.05 (0.97–1.12)	0.215
RP13‐147D17.2	1.07 (0.96–1.2)	0.198	NA	NA
RP5‐828H9.1	24.3 (3.98–148.28)	0.001**	0 (0–1.52)	0.069
SLC35G2	1.31 (1.17–1.47)	0***	1.22 (1.01–1.46)	0.036*
SMAD2	1.33 (1.14–1.55)	0***	1.2 (0.79–1.83)	0.392
SNX7	1.03 (1.01–1.05)	0.002**	0.99 (0.96–1.03)	0.592
TCEB2P2	0.99 (0.93–1.06)	0.739	NA	NA
UGT1A6	1.02 (1.01–1.03)	0.002**	1 (0.97–1.03)	0.96
VTRNA1.2	1.6 (1.12–2.28)	0.009**	1.28 (0.59–2.75)	0.533
ZNF317P1	175.77 (2.87–10756.11)	0.014*	0.1 (0–18296.63)	0.709

*p < 0.05, **p < 0.01, ***p < 0.001.

**FIGURE 5:**
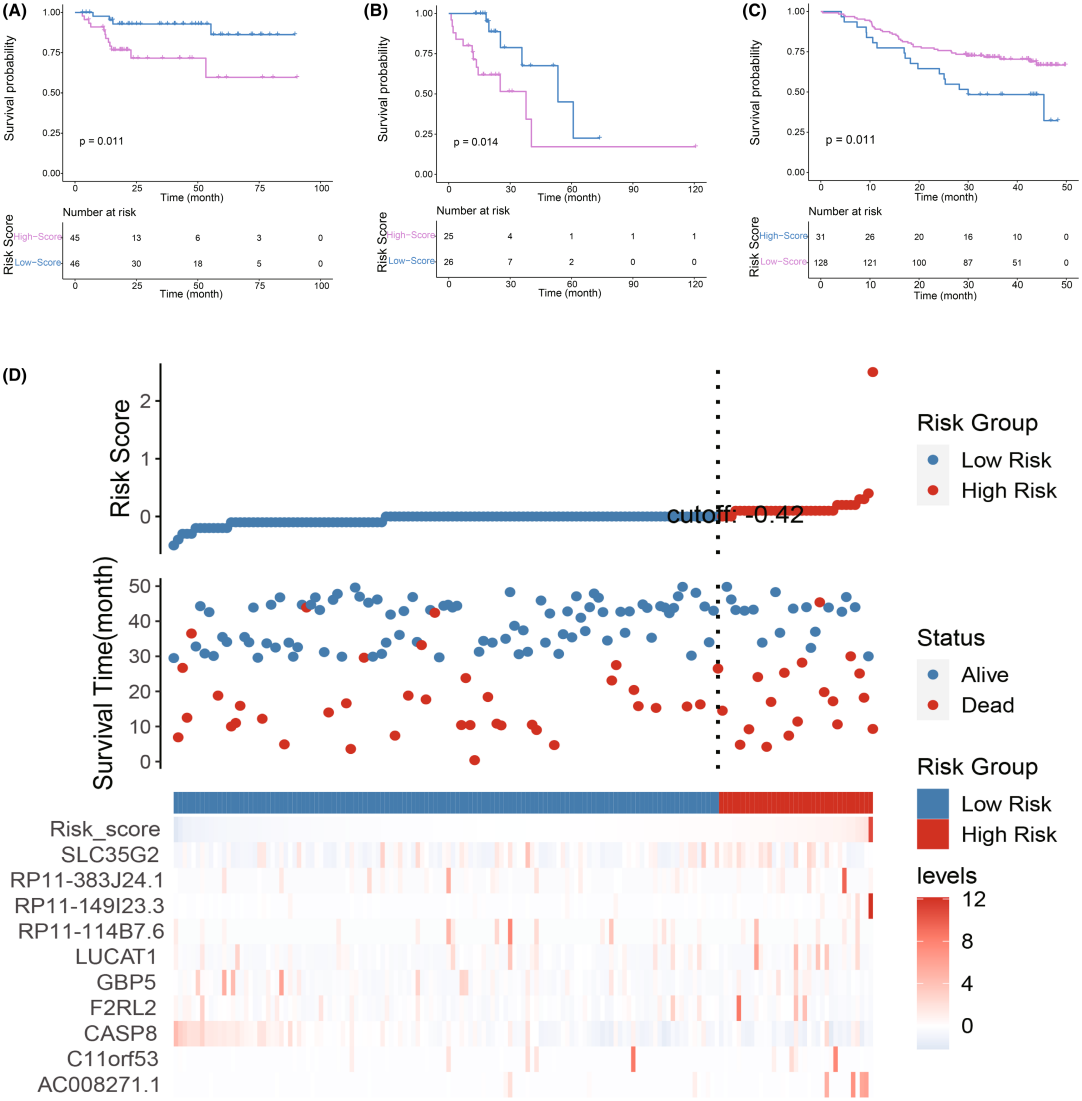
Evaluation of the prognostic value of the risk score signature. (A) K‐M analysis result of the HBV group. (B) K‐M analysis result of the non‐HBV group. (C) K‐M analysis result of the external validation group. (D) Cutoff value (upper panel), survival status (middle panel) and expression levels of the risk score and 10 prognostic MDGs (lower panel) in the external validation group

To confirm the efficacy of this risk score, two internal validation groups from the TCGA‐HCC cohort (HBV group and non‐HBV group) were used. Patients were also separated into high‐ and low‐score groups according to the median values of the risk score. K‐M plotter results suggested that patients in the high‐score group had remarkable poorer prognosis than those in the low‐score group in both the HBV group (Figure 5A) and the non‐HBV group (Figure 5B). In the external validation cohort, the cutoff value was identified by the ROC method. Consistent with previous results, patients in the low‐score group also had a remarkable better prognosis than those in the high‐score group (Figure 5C). Grouping information, survival status, and the expression of the risk score and 10 prognostic MDGs are visualized in Figure 5D.

### 3.4 Clinicopathological features analyses, nomogram construction and evaluation

**FIGURE 6:**
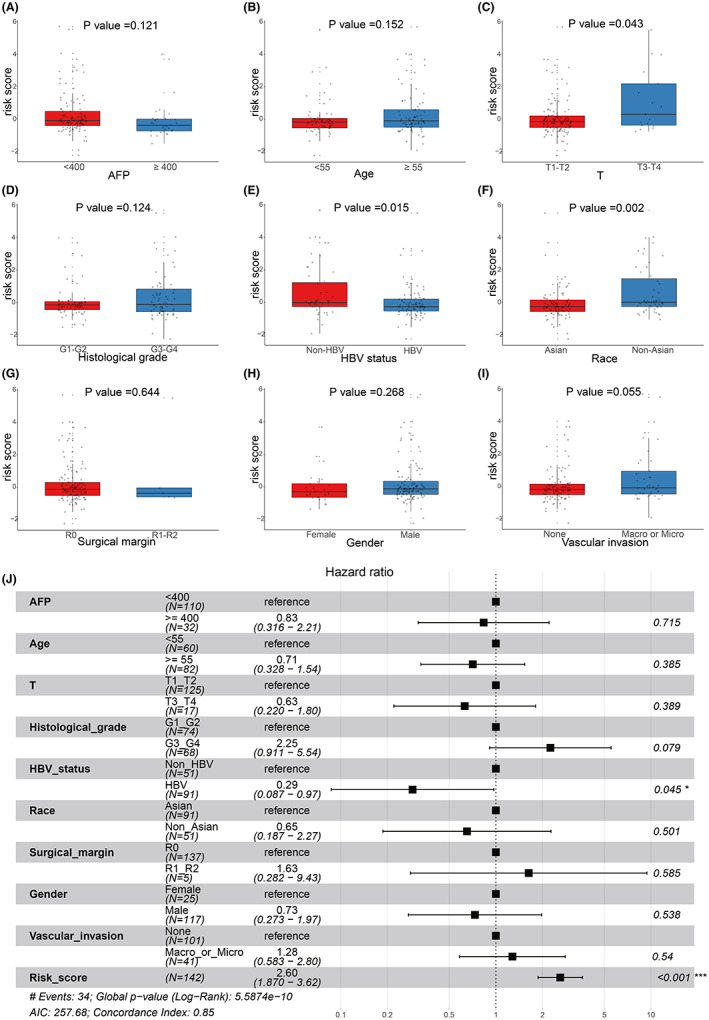
Correlation analysis and clinical subgroup analysis. Boxplot results of the expression levels of risk score among different clinical subgroups including (A) AFP, (B) Age, (C) T stage, (D) Histological grade, (E) HBV status, (F) Race, (G) Surgical margins, (H) Gender and (I) Vascular invasion. (J) Forest plot of clinical features and risk score signature of the TCGA‐HCC cohort

There was no significant difference in clinical information before and after interpolation (Figure S3 and Table S2), so we used the interpolated data for follow‐up analysis. As revealed by the result of univariate Cox analysis, 5 clinicopathological features (HBV status, histological grade, race, T stage and vascular invasion status) significantly related with survival (*p* < 0.05) were selected for multivariate Cox analysis (Table 2). As a result, 2 prognostic clinicopathological features (HBV status and histological grades) (*p* < 0.1) were identified (Table 2). The results of correlation analyses between risk scores and clinicopathological features showed that patients in high‐level T stage and non‐Asian groups had significant higher risk scores, while patients with HBV infection had significant lower risk scores (*p* < 0.05) (Figure 6A–I). To further clarify the impact of different clinical subgroups on prognosis, clinical subgroup analysis was conducted. As shown in Figure 6J, patients with higher histological grades (G3_G4) had a worse prognosis than the patients with lower histological grades (G1_G2), while patients with HBV infection (HBV) had a better prognosis than the patients with non‐HBV infection (non‐HBV).

**TABLE 2:** Univariate and multivariate Cox regression analysis of clinicopathological characteristics and risk score

	Univariate analysis		Multivariate analysis	
Variables	HR (95% CI)	*p*	HR (95% CI)	*p*
AFP	0.81 (0.35–1.86)	0.62	NA	NA
Age	1.03 (0.52–2.02)	0.941	NA	NA
Gender	0.78 (0.34–1.79)	0.559	NA	NA
HBV_status	0.38 (0.19–0.75)	0.005**	0.3 (0.1–0.94)	0.038*
Histological_grade	2.16 (1.07–4.37)	0.032*	2.26 (0.97–5.29)	0.059
Race	2.57 (1.3–5.09)	0.007**	0.67 (0.2–2.24)	0.51
Surgical_margin	3.18 (0.75–13.57)	0.117	NA	NA
T	2.39 (1.04–5.5)	0.041*	0.6 (0.22–1.61)	0.309
Vascular_invasion	2.05 (1.03–4.06)	0.04*	1.26 (0.59–2.71)	0.548
Risk_score	2.72 (2.1–3.51)	0***	2.59 (1.86–3.6)	0***

*p < 0.05, **p < 0.01, ***p < 0.001.

**FIGURE 7:**
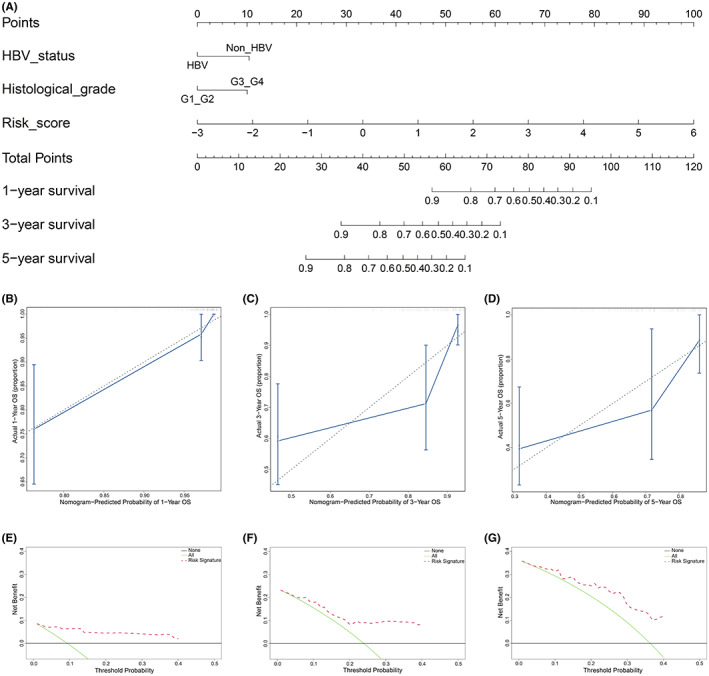
Nomogram building and efficiency assessment. (A) Nomogram for predicting survival probability in HCC. (B‐D) Calibration plots for the nomogram in predicting survival probability. (E‐G) The clinical application efficiencies for predicting survival probability was identified by DCA

According to these results, a prognostic nomogram was constructed by risk score, HBV status and histological grades, and the 1‐, 3‐, and 5‐year OS times of TCGA‐HCC patients were predicted (Figure 7A). Calibration curves (Figure 7B–D) and C index (0.829, 95% confidence interval: 0.794–0.864) confirmed the good prediction efficiency of the nomogram. Subsequently, the clinical application values for predicting OS were identified by DCA (Figure 7E–G).

### 3.5 GSVA and immune analysis

**FIGURE 8:**
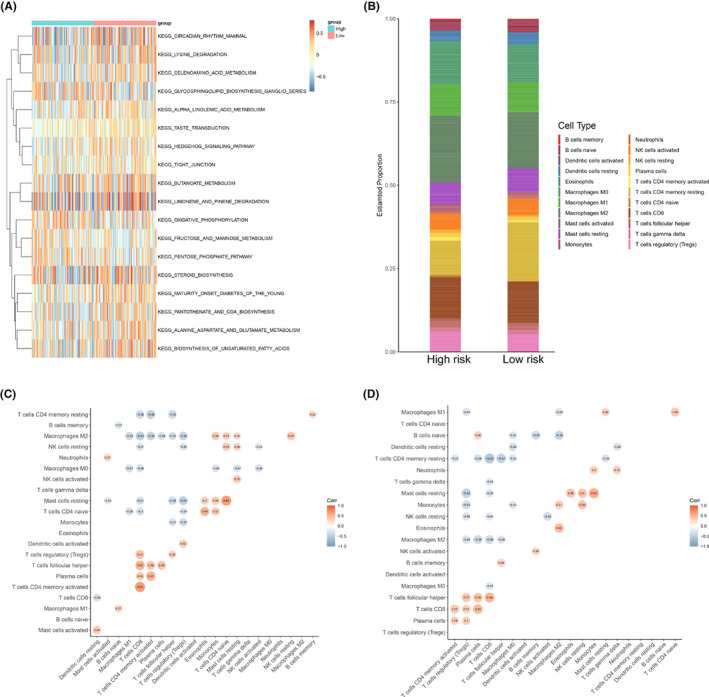
GSVA and immune analysis. (A) GVSA result between the two risk groups in TCGA‐HCC cohort. (B) Analysis of immune cell components. (C, D) Relevance analysis of 21 immune cell types in high‐ (C) and low‐risk (D) score patients

TCGA‐HCC patients were separated into high‐ and low‐score groups according to the median value of the risk score. Nest, GVSA was performed to analyze the differences between these two groups. As revealed by Figure 8A, the most diverse pathways include lysine degradation, selenoamino acid metabolism, the hedgehog signaling pathway, tight junctions, oxidative phosphorylation, etc. To deeply clarify the relationship between the risk signature and immunity, component and correlation analyses of immune cells were conducted using CIBERSOFT in R. As shown in Figure 8B, some immune cells were highly expressed in the high‐score group, such as M0 Macrophages and M2 Macrophages, while other immune cells were highly expressed in the low‐score group, such as naive B cells. In tumor immune microenvironment, immune cells usually interact with each other and play an integral role.^37^ To address the differences of relationships between immune cell types, correlation analyses in the high‐ or low‐score group were performed respectively. As revealed by Figure 8C, there were positive correlations between CD8 T cells and memory activated CD4 T cells or follicular helper T cells, plasma cells and memory activated CD4 T cells, resting mast cells and naive CD4 T cells in the high‐score group (correlation coefficient >0.5). Otherwise, monocytes were positively correlated with resting mast cells, while CD8 T cells were negatively corelated with resting memory CD4 T cells (|correlation coefficient| > 0.5) in the low‐score group (Figure 8D).

## 4 DISCUSSION

The pathological process of HCC develops from hepatitis is different from other causes, such as alcoholic liver disease and NAFLD.^2^, ^38^, ^39^, ^40^ The DNA methylation probe and MDGs signatures have shown a good prediction efficiency of all‐cause HCC and nonviral HCC.^27^, ^28^, ^29^, ^30^ However, whether there is an efficient MDGs signature used to predict hepatitis‐positive HCC remains unclear. In this study, integrated analysis was performed by DNA methylation data and transcription data of HBV/HCV positive patients from the TCGA‐HCC cohort, and 608 DNA methylation‐driver genes were identified. Through random forest screening, as well as subsequent univariate and multivariate Cox progression analysis, 10 methylation‐driver prognostic genes (AC008271.1, C11orf53, CASP8, F2RL2, GBP5, LUCAT1, RP11‐114B7.6, RP11‐149I23.3, RP11‐383 J24.1 and SLC35G2) were screened out and a prognostic risk score signature was constructed. The prognostic value of the risk score signature was validated in the TCGA‐HCC and China‐HCC cohorts. It is noteworthy that methylation‐driver prognostic genes screened from hepatitis‐positive HCC patients are completely different from those from all‐cause HCC and nonviral HCC patients,^28^, ^29^, ^30^ suggesting that DNA methylation plays a role in HCC of different etiologies through different mechanisms.

Among the 10 genes that constructed the risk score, 7 genes (F2RL2, SLC35G2, C11orf53, AC008271.1, RP11‐114B7.6, RP11‐149I23.3 and RP11‐383 J24.1) have never been studied to be associated with HCC. F2RL2 has been reported to be a prognostic marker for glioma and breast cancer.^41^, ^42^ SLC35G2 is a risk factor in clear cell renal cell carcinoma (ccRCC),^43^ while the single nucleotide‐polymorphism (SNP) of C11orf53 can affect the susceptibility to colorectal cancer.^44^, ^45^ Moreover, AC008271.1, RP11‐114B7.6, RP11‐149I23.3 and RP11‐383 J24.1 have never been reported in cancer‐related research. Except for these genes, CASP8, GBP5 and LUCAT1 have been reported to play multiple roles in numerous cancer types, such as head and neck cancer, oral squamous cell carcinoma, esophageal squamous cell carcinoma, colorectal cancer and HCC.^46^, ^47^, ^48^, ^49^, ^50^, ^51^, ^52^, ^53^ For example, recent studies suggested that CASP8 is involved in autophagy‐related apoptosis and can be used as a prognostic marker of HCC together with other molecules,^52^, ^53^ and GBP5 can be used as a prognostic biomarker of HCC together with other genes, and it is closely related to immune characteristics.^54^ Furthermore, LUCAT1 was reported to affecting the proliferation, migration and invasion processes of HCC.^55^, ^56^ However, there is no relevant study on the relationship between these prognostic genes and hepatitis‐positive HCC. In addition, most of these genes have a risk coefficient greater than 0 (AC008271.1, C11orf53, F2RL2, GBP5, LUCAT1, RP11‐149I23.3, RP11‐383 J24.1 and SLC35G2), except for CASP8 and RP11‐114B7.6, suggesting that these genes are adverse prognostic factors in hepatitis‐positive HCC patients. And most of these genes were highly expressed in the high score group, consistent with their coefficients (Figure 4A and Figure 5D).

To further enhance the efficiency of the prognostic model, we included clinicopathological features in the analysis. The results showed that HBV status (*p* = 0.038) and histological grades (*p* = 0.059) were closely related to OS time. Considering that the C index of the prediction nomogram model constructed only by risk score and HBV status (C index: 0.794, 95% confidence interval: 0.748–0.84) was lower than that constructed by risk score, HBV status and histological grades (C index: 0.829, 95% confidence interval: 0.794–0.864), and high histological grades have been widely accepted to be related with adverse prognosis of many cancers, a prediction nomogram was constructed by risk score, HBV status and histological grades in this study.

Immunotherapy is a promising tumor therapeutic method targeting a series of solid and nonsolid tumors.^5^, ^57^, ^58^, ^59^ Although immunotherapeutic drugs combined with angiogenesis inhibitors have shown better efficacy than the methods recommended in previous guidelines in advanced unresectable HCC, the prognosis is still unsatisfactory.^60^ Therefore, it is urgent to systematically clarify the immune heterogeneity of HCC, so as to find synergistic targets to enhance the efficacy of immunotherapy. In this study, the results of component analysis revealed that the proportions of immune cells were significantly different between the high‐ and low‐score groups, as were the interaction relationships between immune cells in the two groups. These results indicate that different immunotherapy approaches may be applicable to the different types of patients divided by the risk score constructed in our study. For example, M2 Macrophages, classic immunosuppressive cells, were highly expressed in high‐score group, suggesting that reversing the immunosuppressive environment of the high‐score group may improve the efficacy of immunotherapy. In addition, resting memory CD4 T cells were highly expressed in low‐score group, and they were negatively corelated with CD8 T cells, suggesting that targeting resting memory CD4 T cells may be an important synergistic strategy for immunotherapy in patients with HCC in the low‐score group.

However, there are some limitations in the present study. First, the risk score was constructed by RNA‐sequencing data, and some of these 10 prognostic genes cannot be detected in many major microarray platforms, which limits its validation sample size and application range. Second, the relationship between the risk score and immunotherapy only preliminarily explored in this study, which needs to be further clarified through experimental research and clinical trials.

In conclusion, our study explored a DNA methylation‐driver gene risk score signature and an efficient nomogram for the survival prediction of hepatitis‐positive HCC patients. In addition, the differences of immune characteristics between different risk groups were preliminarily clarified, which will guide the follow‐up exploration of improving the efficacy of immunotherapy for HCC.

## CONFLICT OF INTEREST

None.

## AUTHORS’ CONTRIBUTIONS

Jie Fu and Xundi Xu conceived this study and analyzed the data. Wei Qin, Qing Tong, Zhenghao Li, Yaoli Shao, Zhiqiang Liu, Chun Liu and Zicheng Wang obtained the datasets from online databases. All authors contributed to the manuscript.

## ETHICS STATEMENT

Not applicable.

## Supporting information


Figure S1

Figure S2

Figure S3

Table S1

TABLE S2

## Data Availability

The datasets analyzed in this study are available in the TCGA and NODE, https: //cancergeno me.nih.gov/ and https://www.biosino.org/node.

## References

[1] Sung H , Ferlay J , Siegel RL , et al. Global cancer statistics 2020: GLOBOCAN estimates of incidence and mortality worldwide for 36 cancers in 185 countries. CA Cancer J Clin. 2021;71(3):209‐249. 33538338 10.3322/caac.21660

[2] Terry Cheuk‐Fung Y , Hye Won L , Wah Kheong C , Grace Lai‐Hung W , Vincent W‐SW . Asian perspective on NAFLD‐associated HCC. J Hepatol. 2022;76(3):726‐734. 34619251 10.1016/j.jhep.2021.09.024

[3] Mallet V , Parlati L , Martinino A , et al. Burden of liver disease progression in hospitalized patients with type 2 diabetes mellitus. J Hepatol. 2021;76:265‐274. 34606913 10.1016/j.jhep.2021.09.030

[4] Ren Z , Xu J , Bai Y , et al. Sintilimab plus a bevacizumab biosimilar (IBI305) versus sorafenib in unresectable hepatocellular carcinoma (ORIENT‐32): a randomised, open‐label, phase 2‐3 study. Lancet Oncol. 2021;22(7):977‐990. 34143971 10.1016/S1470-2045(21)00252-7

[5] Galle PR , Finn RS , Qin S , et al. Patient‐reported outcomes with atezolizumab plus bevacizumab versus sorafenib in patients with unresectable hepatocellular carcinoma (IMbrave150): an open‐label, randomised, phase 3 trial. Lancet Oncol. 2021;22(7):991‐1001. 34051880 10.1016/S1470-2045(21)00151-0

[6] Scheiner B , Pomej K , Kirstein MM , et al. Prognosis of patients with hepatocellular carcinoma treated with immunotherapy ‐ development and validation of the CRAFITY score. J Hepatol. 2022;76(2):353‐363. 34648895 10.1016/j.jhep.2021.09.035

[7] Zhao S , Allis CD , Wang GG . The language of chromatin modification in human cancers. Nat Rev Cancer. 2021;21(7):413‐430. 34002060 10.1038/s41568-021-00357-x PMC10507815

[8] Zhang Z , Zhou J , Tan P , et al. Epigenomic diversity of cortical projection neurons in the mouse brain. Nature. 2021;598(7879):167‐173. 34616065 10.1038/s41586-021-03223-w PMC8494636

[9] Janssen SM , Lorincz MC . Interplay between chromatin marks in development and disease. Nat Rev Genetics. 2021;23:137‐153. 34608297 10.1038/s41576-021-00416-x

[10] Min JL , Hemani G , Hannon E , et al. Genomic and phenotypic insights from an atlas of genetic effects on DNA methylation. Nat Genet. 2021;53(9):1311‐1321. 34493871 10.1038/s41588-021-00923-x PMC7612069

[11] Liu J , Ji C , Wang Y , Zhang C , Zhu H . Identification of methylation‐driven genes prognosis signature and immune microenvironment in uterus corpus endometrial cancer. Cancer Cell Int. 2021;21(1):365. 34246261 10.1186/s12935-021-02038-z PMC8272318

[12] Cai GX , Cai M , Feng Z , Liu R , Liang L , Zhou P . A multilocus blood‐based assay targeting circulating tumor DNA methylation enables early detection and early relapse prediction of colorectal cancer. Gastroenterology. 2021;161:2053‐2056.e2. 34487783 10.1053/j.gastro.2021.08.054

[13] Pan H , Renaud L , Chaligne R , et al. Discovery of candidate DNA methylation cancer driver genes. Cancer Discov. 2021;11(9):2266‐2281. 33972312 10.1158/2159-8290.CD-20-1334 PMC8419066

[14] Kumaraswamy A , Welker Leng KR , Westbrook TC , et al. Recent advances in epigenetic biomarkers and epigenetic targeting in prostate cancer. Eur Urol. 2021;80(1):71‐81. 33785255 10.1016/j.eururo.2021.03.005 PMC8547521

[15] Jang M , An J , Oh SW , et al. Matrix stiffness epigenetically regulates the oncogenic activation of the yes‐associated protein in gastric cancer. Nat Biomed Eng. 2021;5(1):114‐123. 33288878 10.1038/s41551-020-00657-x

[16] Moss J , Zick A , Grinshpun A , et al. Circulating breast‐derived DNA allows universal detection and monitoring of localized breast cancer. Anna Oncol. 2020;31(3):395‐403. 10.1016/j.annonc.2019.11.014 32067681

[17] Robertson AG , Shih J , Yau C , et al. Integrative analysis identifies four molecular and clinical subsets in uveal melanoma. Cancer Cell. 2017;32(2):204‐20.e15. 28810145 10.1016/j.ccell.2017.07.003 PMC5619925

[18] Hou P , Bao S , Fan D , et al. Machine learning‐based integrative analysis of methylome and transcriptome identifies novel prognostic DNA methylation signature in uveal melanoma. Brief Bioinform. 2021;22(4):bbaa371. 33367533 10.1093/bib/bbaa371

[19] Guo Y , Mao X , Qiao Z , Chen B , Jin F . A novel promoter CpG‐based signature for long‐term survival prediction of breast cancer patients. Front Oncol. 2020;10:579692. 33194705 10.3389/fonc.2020.579692 PMC7606941

[20] Xu Z , Gujar H , Fu G , et al. A novel DNA methylation signature as an independent prognostic factor in muscle‐invasive bladder cancer. Front Oncol. 2021;11:614927. 33659216 10.3389/fonc.2021.614927 PMC7917237

[21] Wong CC , Xu J , Bian X , et al. In colorectal cancer cells with mutant KRAS, SLC25A22‐mediated Glutaminolysis reduces DNA demethylation to increase WNT signaling, stemness, and drug resistance. Gastroenterology. 2020;159(6):2163‐80.e6. 32814111 10.1053/j.gastro.2020.08.016

[22] Ding X , He M , Chan AWH , et al. Genomic and epigenomic features of primary and recurrent hepatocellular carcinomas. Gastroenterology. 2019;157(6):1630‐1645.e6. 31560893 10.1053/j.gastro.2019.09.005

[23] Meunier L , Hirsch TZ , Caruso S , et al. DNA methylation signatures reveal the diversity of processes remodeling hepatocellular carcinoma methylomes. Hepatology (Baltimore, Md). 2021;74(2):816‐834. 10.1002/hep.31796 PMC12452108 33713365

[24] Yan Q , Zhang Y , Fang X , et al. PGC7 promotes tumor oncogenic dedifferentiation through remodeling DNA methylation pattern for key developmental transcription factors. Cell Death Differ. 2021;28(6):1955‐1970. 33500560 10.1038/s41418-020-00726-3 PMC8185079

[25] Nagaraju GP , Dariya B , Kasa P , Peela S , El‐Rayes BF . Epigenetics in hepatocellular carcinoma. Semin Cancer Biol. 2021;S1044‐579X(21):00211‐X. 10.1016/j.semcancer.2021.07.017 34324953

[26] Liu A , Wu Q , Peng D , et al. A novel strategy for the diagnosis, prognosis, treatment, and chemoresistance of hepatocellular carcinoma: DNA methylation. Med Res Rev. 2020;40(5):1973‐2018. 32525219 10.1002/med.21696

[27] Zhu L , Guo W . Combined DNA methylation and transcriptomic assessments to determine a prognostic model for PD‐1‐negative hepatocellular carcinoma. Front Cell Dev Biol. 2021;9:708819. 34458266 10.3389/fcell.2021.708819 PMC8385720

[28] Li GX , Ding ZY , Wang YW , et al. Integrative analysis of DNA methylation and gene expression identify a six epigenetic driver signature for predicting prognosis in hepatocellular carcinoma. J Cell Physiol. 2019;234(7):11942‐11950. 30536816 10.1002/jcp.27882

[29] Chen J , Wang X , Wang X , et al. A FITM1‐related methylation signature predicts the prognosis of patients with non‐viral hepatocellular carcinoma. Front Genet. 2020;11:99. 32174969 10.3389/fgene.2020.00099 PMC7056874

[30] Xu Q , Hu Y , Chen S , et al. Immunological significance of prognostic DNA methylation sites in hepatocellular carcinoma. Front Mol Biosci. 2021;8:683240. 34124163 10.3389/fmolb.2021.683240 PMC8187884

[31] Gao Q , Zhu H , Dong L , et al. Integrated proteogenomic characterization of HBV‐related hepatocellular carcinoma. Cell. 2019;179(2):561‐77.e22. 31585088 10.1016/j.cell.2019.08.052

[32] Morris TJ , Butcher LM , Feber A , et al. ChAMP: 450k Chip analysis methylation pipeline. Bioinformatics (Oxford, England). 2014;30(3):428‐430. 24336642 10.1093/bioinformatics/btt684 PMC3904520

[33] Gevaert O . MethylMix: an R package for identifying DNA methylation‐driven genes. Bioinformatics (Oxford, England). 2015;31(11):1839‐1841. 25609794 10.1093/bioinformatics/btv020 PMC4443673

[34] Yu G , Wang LG , Han Y , He QY . clusterProfiler: an R package for comparing biological themes among gene clusters. Omics: J Integr Biol. 2012;16(5):284‐287. 10.1089/omi.2011.0118 PMC3339379 22455463

[35] Van Belle V , Pelckmans K , Van Huffel S , Suykens JA . Improved performance on high‐dimensional survival data by application of survival‐SVM. Bioinformatics (Oxford, England). 2011;27(1):87‐94. 21062763 10.1093/bioinformatics/btq617

[36] Hänzelmann S , Castelo R , Guinney J . GSVA: gene set variation analysis for microarray and RNA‐seq data. BMC Bioinformatics. 2013;14:7. 23323831 10.1186/1471-2105-14-7 PMC3618321

[37] Llovet JM , Castet F , Heikenwalder M , et al. Immunotherapies for hepatocellular carcinoma. Nat Rev Clin Oncol. 2021;19:151‐172. 34764464 10.1038/s41571-021-00573-2

[38] Sekiba K , Otsuka M , Funato K , et al. HBx‐induced degradation of Smc5/6 complex impairs homologous recombination‐mediated repair of damaged DNA. J Hepatol. 2021;76:53‐62. 34478763 10.1016/j.jhep.2021.08.010

[39] Kumar A , Hossain RA , Yost SA , et al. Structural insights into hepatitis C virus receptor binding and entry. Nature. 2021;598:521‐525. 34526719 10.1038/s41586-021-03913-5 PMC8542614

[40] Ng SWK , Rouhani FJ , Brunner SF , et al. Convergent somatic mutations in metabolism genes in chronic liver disease. Nature. 2021;598:473‐478. 34646017 10.1038/s41586-021-03974-6

[41] Liu S , Song A , Wu Y , et al. Analysis of genomics and immune infiltration patterns of epithelial‐mesenchymal transition related to metastatic breast cancer to bone. Transl Oncol. 2021;14(2):100993. 33333372 10.1016/j.tranon.2020.100993 PMC7736716

[42] Lvu W , Fei X , Chen C , Zhang B . In silico identification of the prognostic biomarkers and therapeutic targets associated with cancer stem cell characteristics of glioma. Biosci Rep. 2020;40(8):BSR20201037. 32725165 10.1042/BSR20201037 PMC7418212

[43] Wrzesiński T , Szelag M , Cieślikowski WA , et al. Expression of pre‐selected TMEMs with predicted ER localization as potential classifiers of ccRCC tumors. BMC Cancer. 2015;15:518. 26169495 10.1186/s12885-015-1530-4 PMC5015219

[44] Biancolella M , Fortini BK , Tring S , et al. Identification and characterization of functional risk variants for colorectal cancer mapping to chromosome 11q23.1. Hum Mol Genet. 2014;23(8):2198‐2209. 24256810 10.1093/hmg/ddt584 PMC3959808

[45] Closa A , Cordero D , Sanz‐Pamplona R , et al. Identification of candidate susceptibility genes for colorectal cancer through eQTL analysis. Carcinogenesis. 2014;35(9):2039‐2046. 24760461 10.1093/carcin/bgu092 PMC4146415

[46] Feng B , Shen Y , Pastor Hostench X , et al. Integrative analysis of multi‐omics data identified EGFR and PTGS2 as key nodes in a gene regulatory network related to immune phenotypes in head and neck cancer. Clin Cancer Res. 2020;26(14):3616‐3628. 32161122 10.1158/1078-0432.CCR-19-3997

[47] Prime SS , Cirillo N , Cheong SC , Prime MS , Parkinson EK . Targeting the genetic landscape of oral potentially malignant disorders has the potential as a preventative strategy in oral cancer. Cancer Lett. 2021;518:102‐114. 34139286 10.1016/j.canlet.2021.05.025

[48] Yu TT , Sang XY , Han N , et al. Macrophages mediated delivery of chlorin e6 and treatment of lung cancer by photodynamic reprogramming. Int Immunopharmacol. 2021;100:108164. 34562845 10.1016/j.intimp.2021.108164

[49] Liu PF , Shu CW , Lee CH , et al. Clinical significance and the role of guanylate‐binding protein 5 in Oral squamous cell carcinoma. Cancer. 2021;13(16):4043. 10.3390/cancers13164043 PMC8394330 34439200

[50] Wu R , Li L , Bai Y , et al. The long noncoding RNA LUCAT1 promotes colorectal cancer cell proliferation by antagonizing Nucleolin to regulate MYC expression. Cell Death Dis. 2020;11(10):908. 33097685 10.1038/s41419-020-03095-4 PMC7584667

[51] Yoon JH , You BH , Park CH , Kim YJ , Nam JW , Lee SK . The long noncoding RNA LUCAT1 promotes tumorigenesis by controlling ubiquitination and stability of DNA methyltransferase 1 in esophageal squamous cell carcinoma. Cancer Lett. 2018;417:47‐57. 29247823 10.1016/j.canlet.2017.12.016

[52] Brun S , Bestion E , Raymond E , et al. GNS561, a clinical‐stage PPT1 inhibitor, is efficient against hepatocellular carcinoma via modulation of lysosomal functions. Autophagy. 2022;18(3):678‐694. 34740311 10.1080/15548627.2021.1988357 PMC9037544

[53] Zheng S , Xie X , Guo X , et al. Identification of a Pyroptosis‐related gene signature for predicting overall survival and response to immunotherapy in hepatocellular carcinoma. Front Genet. 2021;12:789296. 34925465 10.3389/fgene.2021.789296 PMC8678488

[54] Xiang S , Li J , Shen J , et al. Identification of prognostic genes in the tumor microenvironment of hepatocellular carcinoma. Front Immunol. 2021;12:653836. 33897701 10.3389/fimmu.2021.653836 PMC8059369

[55] Xing C , Sun SG , Yue ZQ , Bai F . Role of lncRNA LUCAT1 in cancer. Biomed Pharmacother. 2021;134:111158. 33360049 10.1016/j.biopha.2020.111158

[56] Lou Y , Yu Y , Xu X , et al. Long non‐coding RNA LUCAT1 promotes tumourigenesis by inhibiting ANXA2 phosphorylation in hepatocellular carcinoma. J Cell Mol Med. 2019;23(3):1873‐1884. 30588744 10.1111/jcmm.14088 PMC6378214

[57] Dummer R , Gyorki DE , Hyngstrom J , et al. Neoadjuvant talimogene laherparepvec plus surgery versus surgery alone for resectable stage IIIB‐IVM1a melanoma: a randomized, open‐label, phase 2 trial. Nat Med. 2021;27(10):1789‐1796. 34608333 10.1038/s41591-021-01510-7

[58] Spiegel JY , Patel S , Muffly L , et al. CAR T cells with dual targeting of CD19 and CD22 in adult patients with recurrent or refractory B cell malignancies: a phase 1 trial. Nat Med. 2021;27(8):1419‐1431. 34312556 10.1038/s41591-021-01436-0 PMC8363505

[59] Topp BG , Thiagarajan K , De Alwis DP , Snyder A , Hellmann MD . Lesion‐level heterogeneity of radiologic progression in patients treated with pembrolizumab. Ann Oncol. 2021;32:1618‐1625. 34543717 10.1016/j.annonc.2021.09.006

[60] Finn RS , Qin S , Ikeda M , et al. Atezolizumab plus Bevacizumab in Unresectable Hepatocellular Carcinoma. N Engl J Med. 2020;382(20):1894‐1905. 32402160 10.1056/NEJMoa1915745

