# Supplementary figures and images for: A novel DNA methylation‐driver gene signature for long‐term survival prediction of hepatitis‐positive hepatocellular carcinoma patients
Source: Cancer Med. 2022 May 30;11(23):4721–35. doi: 10.1002/cam4.4838 (PMC9741990; doi:10.1002/cam4.4838)

(A)

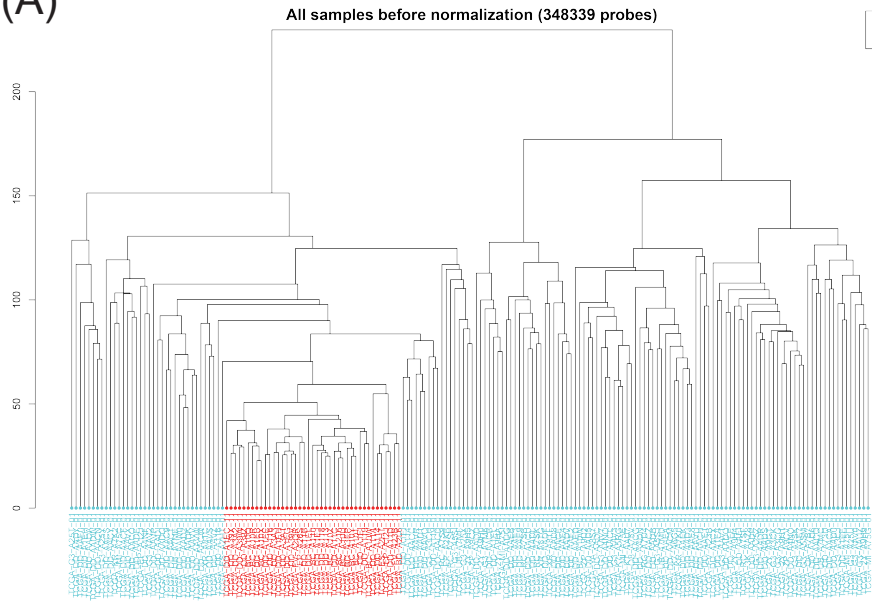

(B)

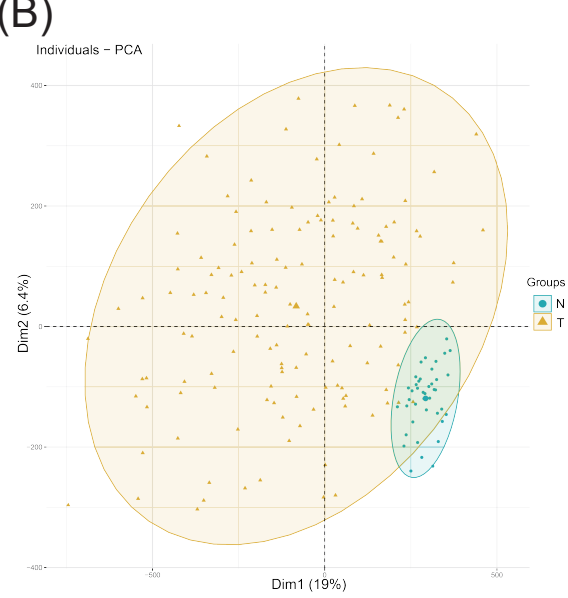

Supplement: Supplementary file 1 — Figure S1 [file CAM4-11-4721-s002.pdf]

(A)

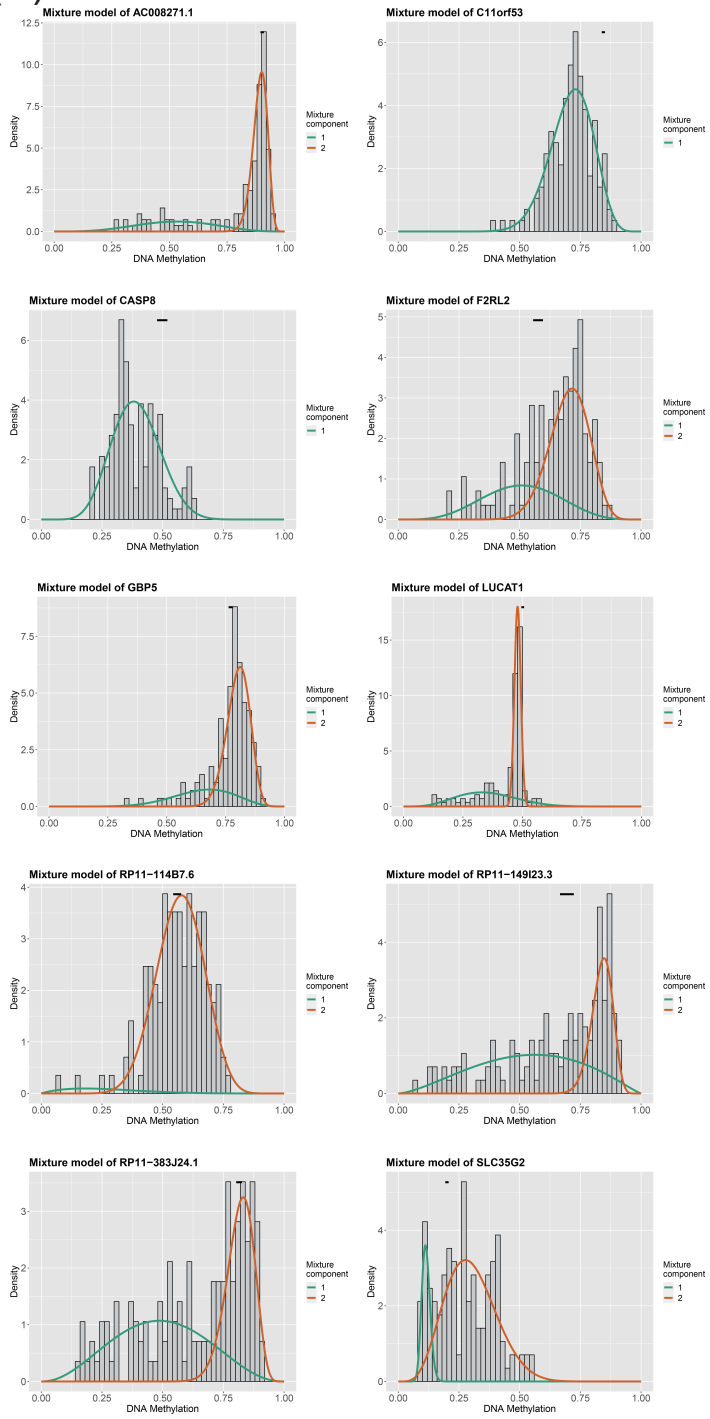

(B)

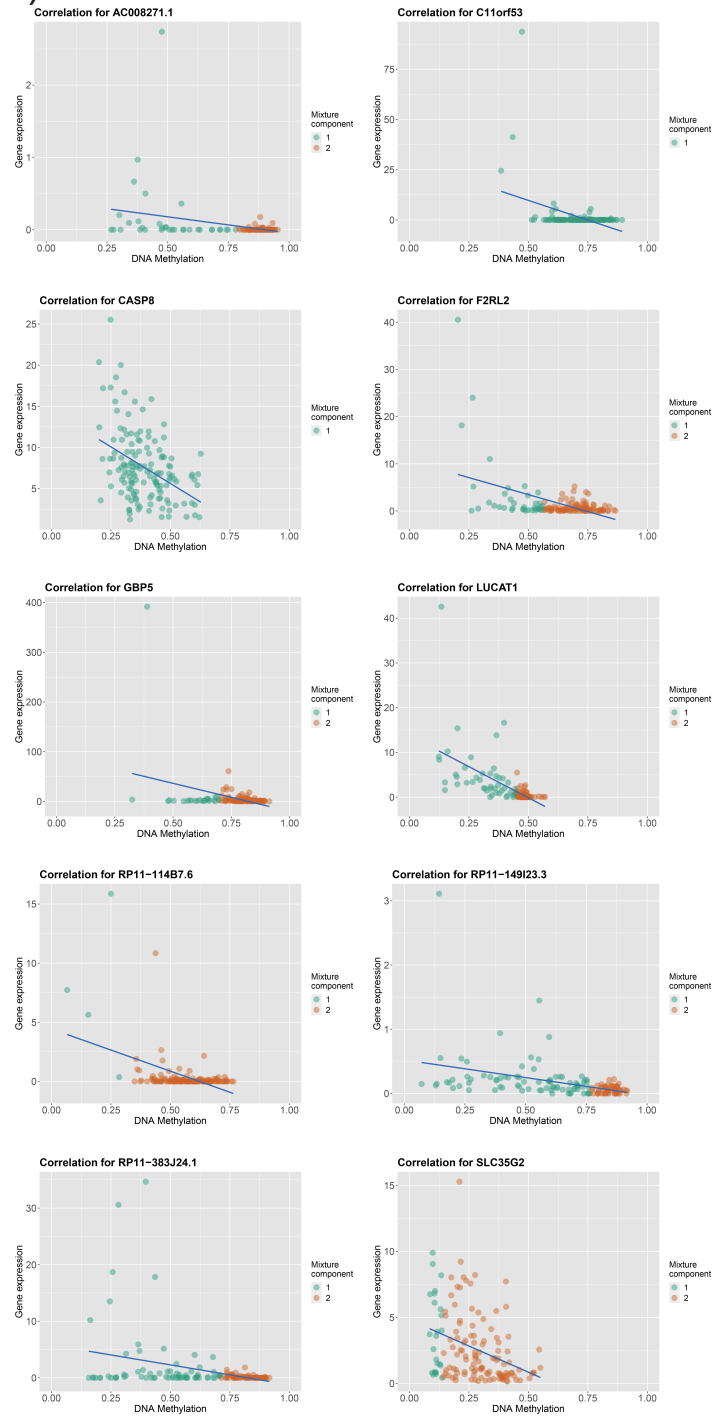

Supplement: Supplementary file 2 — Figure S2 [file CAM4-11-4721-s005.pdf]

(A)

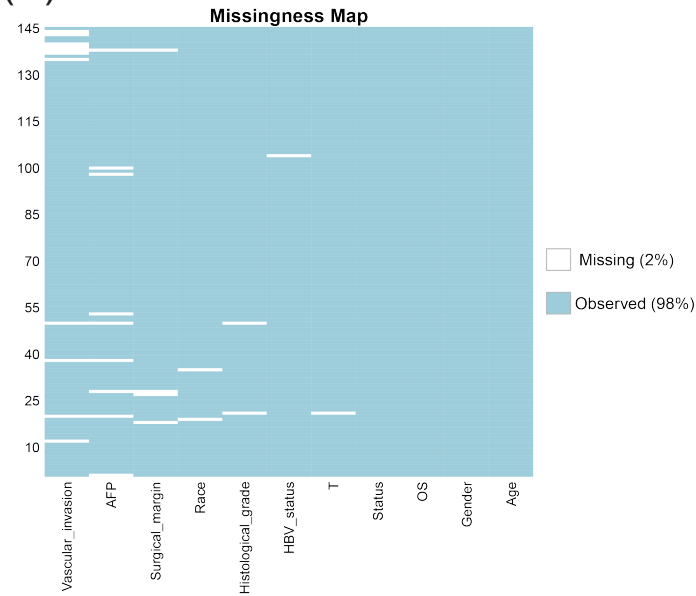

(B)

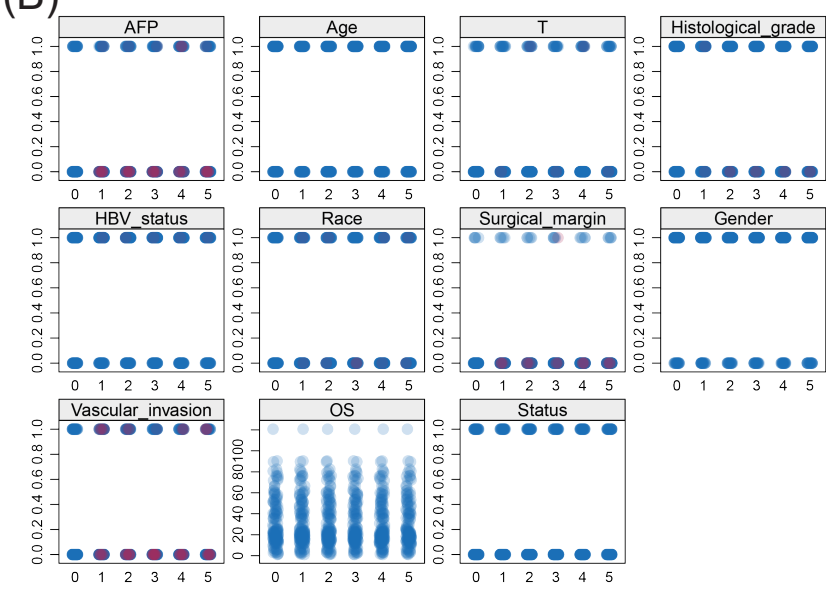

Supplement: Supplementary file 3 — Figure S3 [file CAM4-11-4721-s003.pdf]
