# Supplementary material for: A novel DNA methylation‐driver gene signature for long‐term survival prediction of hepatitis‐positive hepatocellular carcinoma patients
Source: Cancer Med. 2022 May 30;11(23):4721–35. doi: 10.1002/cam4.4838 (PMC9741990; doi:10.1002/cam4.4838)
Supplement: Supplementary file 5 — TABLE S2 [file CAM4-11-4721-s004.docx]

TABLE S2 Comparison of clinicopathological characteristics before and after interpolation.

|  | Before-interpolation (No.) | After-interpolation (No.) | P |
| --- | --- | --- | --- |
| Variables | 142 | 142 |  |
| AFP (mean(sd)) | 0.23 (0.42) | 0.22 (0.42) | 0.89 |
| Age (mean(sd)) | 0.57 (0.5) | 0.57 (0.5) | 1 |
| T (mean(sd)) | 0.12 (0.33) | 0.12 (0.32) | 0.98 |
| Histological_grade (mean(sd)) | 0.49 (0.5) | 0.49 (0.5) | 1 |
| HBV_status (mean(sd)) | 0.65 (0.48) | 0.65 (0.48) | 0.97 |
| Race (mean(sd)) | 0.35 (0.48) | 0.35 (0.48) | 0.97 |
| Surgical_margin (mean(sd)) | 0.04 (0.19) | 0.03 (0.18) | 0.97 |
| Gender (mean(sd)) | 0.83 (0.38) | 0.83 (0.38) | 1 |
| Vascular_invasion (mean(sd)) | 0.28 (0.45) | 0.29 (0.46) | 0.8 |
| OS (mean(sd)) | 30.55 (22.52) | 30.55 (22.52) | 1 |
| Status (mean(sd)) | 0.23 (0.43) | 0.23 (0.43) | 1 |

Group information: AFP level (“< 400” = 0, “≥ 400” = 1), Age (“< 55” = 0, “≥ 55” = 1), T stage (“T1-T2” = 0, “T3-T4” = 1), Histological grade (“G1-G2” = 0, “G3-G4” =1), HBV status (“HBV” = 1, “non-HBV” = 0), Race (“Asian” = 0, “non-Asian” = 1), Surgical margin (“R0” = 0, “R1-R2” = 1), Gender (“female” = 0, “male” = 1) and Vascular invasion (“None” = 0, “Macro-Micro” =1 ), Status (“Alive” = 0, “Dead” = 1).
